# Accessing 3D Printed Vascular Phantoms for Procedural Simulation

**DOI:** 10.3389/fsurg.2020.626212

**Published:** 2021-01-27

**Authors:** Jasamine Coles-Black, Damien Bolton, Jason Chuen

**Affiliations:** ^1^3dMedLab, Austin Health, The University of Melbourne, Parkville, VIC, Australia; ^2^Department of Surgery, Austin Health, The University of Melbourne, Melbourne, VIC, Australia

**Keywords:** 3D printing, vascular phantom, simulation, fluoroscopy, angiography, AAA (abdominal aortic aneurysm), EVAR, FEVAR

## Abstract

**Introduction:** 3D printed patient-specific vascular phantoms provide superior anatomical insights for simulating complex endovascular procedures. Currently, lack of exposure to the technology poses a barrier for adoption. We offer an accessible, low-cost guide to producing vascular anatomical models using routine CT angiography, open source software packages and a variety of 3D printing technologies.

**Methods:** Although applicable to all vascular territories, we illustrate our methodology using Abdominal Aortic Aneurysms (AAAs) due to the strong interest in this area. CT aortograms acquired as part of routine care were converted to representative patient-specific 3D models, and then printed using a variety of 3D printing technologies to assess their material suitability as aortic phantoms. Depending on the technology, phantoms cost $20–$1,000 and were produced in 12–48 h. This technique was used to generate hollow 3D printed thoracoabdominal aortas visible under fluoroscopy.

**Results:** 3D printed AAA phantoms were a valuable addition to standard CT angiogram reconstructions in the simulation of complex cases, such as short or very angulated necks, or for positioning fenestrations in juxtarenal aneurysms. Hollow flexible models were particularly useful for device selection and in planning of fenestrated EVAR. In addition, these models have demonstrated utility other settings, such as patient education and engagement, and trainee and anatomical education. Further study is required to establish a material with optimal cost, haptic and fluoroscopic fidelity.

**Conclusion:** We share our experiences and methodology for developing inexpensive 3D printed vascular phantoms which despite material limitations, successfully mimic the procedural challenges encountered during live endovascular surgery. As the technology continues to improve, 3D printed vascular phantoms have the potential to disrupt how endovascular procedures are planned and taught.

## Introduction

### 3D Printed Vascular Phantoms

3D printing is a manufacturing technique which has gained attention in surgery recently as a means of rapidly producing patient-specific anatomical models for the purposes of procedural simulation and training. This accessible technology allows imaging to be converted into physical, patient-specific models within the hospital setting, enabling surgeons and other proceduralists to rapidly access true-to-scale representations of patient anatomy for superior visualization and planning.

3D printing is anticipated to represent the next step in personalized medicine. Despite the nascence of the technology, its utility as a tool in presurgical planning and intraoperative visualization is currently being examined via clinical trials (1). In addition, its potential in other settings is being explored, such as for patient education and engagement and trainee education. Due to the expiry of patents leading to the democratization of 3D printing technology, desktop 3D printers are now in the price range of office paper printers, well within reach of all surgical departments seeking to produce of patient-specific 3D printed anatomical models in-house. In the field of vascular intervention, 3D printed models have been used as presurgical simulation tools in the planning of Endovascular Aneurysm Repair (EVAR) (2), and complex endovascular aortic techniques such as fenestrated or branched EVAR (3, 4). 3D printed vascular models have also been explored in procedural simulation involving other vascular territories, such as coil embolisation of cerebral aneurysms (5) or splenic artery aneurysms (6).

**Figure 1:**
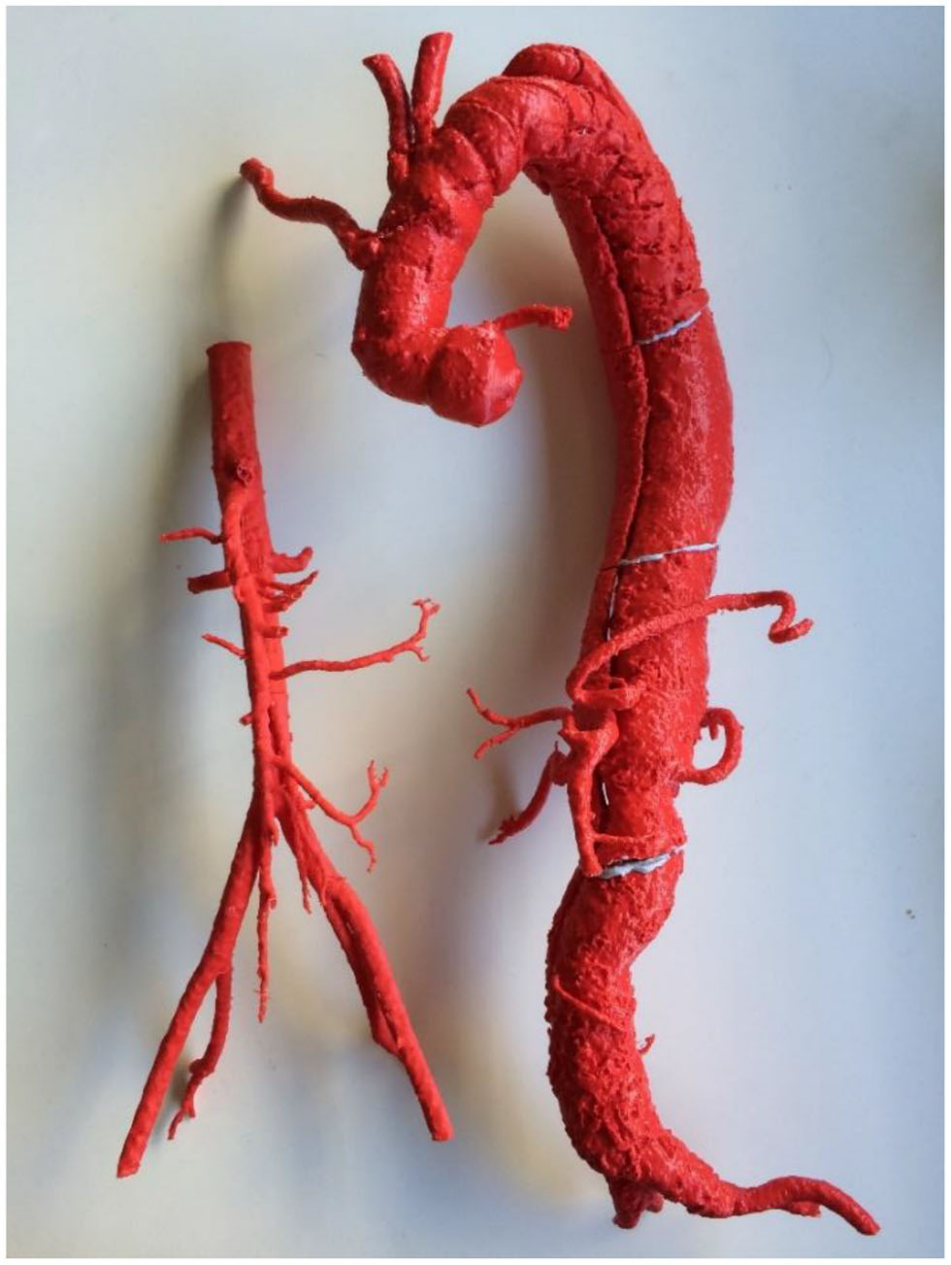
A 3D printed complex Type B aortic dissection for reintervention (right) compared to normal aorta (left). Both models were 3D printed using FDM technology and ABS filament. Note that due to limitations in the size of the print bed, the Type B aortic dissection was printed in four pieces.

Our frontier experiences with the technology mirror those of other groups, with 3D printed patient-specific vascular models providing superior anatomical insights for simulating complex procedures (Figure 1). This has clear advantages regarding patient safety with reduced time under anesthesia, reduction in operation time (7), shorter recovery times, and a reduction in blood loss intraoperatively (8), resulting in cost savings to health services. However, despite the promise of 3D printed patient-specific phantoms for simulation, lack of exposure to the technology amongst vascular proceduralists poses a barrier for adoption (9).

We offer an accessible, low-cost guide to producing vascular anatomical models using routine CT angiography, open source software packages and a variety of 3D printing technologies, with a focus on Abdominal Aortic Aneurysms (AAAs) to showcase its utility. However, the techniques described are equally applicable to any vascular territory imaged using Computed Tomographic (CT) angiography.

### Abdominal Aortic Aneurysms

An Abdominal Aortic Aneurysm is a dilatation of the abdominal aorta to at least 1.5 times its usual size, or an outer diameter of 3 cm (10). As a true aneurysm, the dilatation affects all three layers of the aortic wall, namely the intima, media, and adventitia. AAAs cause a substantial burden on the healthcare system. Rupture results in death in 65% of cases, with a perioperative mortality of 32% (11). In addition, ruptured AAAs are responsible for 1.3% of total deaths in 65–85 year old males in developed countries (10).

The infrarenal aorta is the most common site of AAA formation, and the most favorable anatomical morphology for repair, either via an endovascular approach with EVAR or open surgical repair via infrarenal aortic clamping. Contemporary endovascular technology has made great strides, with EVAR seen as standard care in patients with appropriate anatomy (12).

However, when faced with patient anatomy beyond the standard infrarenal AAA, the endovascular surgeon must modify his or her approach to these complex cases. If the aneurysm extends to but does not involve the renal artery ostia, it is considered a juxtarenal AAA. If the aneurysm extends further superiorly involving the renal arteries and visceral arteries, it is termed a pararenal or paravisceral AAA. Endovascular repair of these complex anatomies is challenging, requiring branched or fenestrated stent grafts, or may be technically unfeasible with current levels of technology.

### Utility of 3D Printed Phantoms in AAA Simulation

As Vascular Surgery experiences a fundamental shift with increasing endovascular and decreasing open repairs, the reach of EVAR increasingly extends to patients with complex anatomy outside of the standard infrarenal AAA, adding to the complexity of contemporary AAA repair.

Well-known potential complications of EVAR include endoleak, graft occlusion, migration or infection (13, 14), requiring further endovascular or open surgical revision and further costs incurred to the healthcare system. As such, adequate visualization of the patient's unique anatomy, appropriate graft selection, the ability to predict intraoperative difficulties and the shape of the graft after deployment are paramount to the improvement of this young technique, and the cost to the public health system. Many of these complications would be mitigated with the opportunity to simulate the proposed procedure and select devices using patient-specific AAA phantoms.

In addition, there is a growing role for 3D printed EVAR simulators in training the next generation of Vascular Surgeons (7). The benefits of simulation in procedural training have been well-described in the Vascular Surgery literature (15, 16). Simulation allows for a “dry run,” improving trainee confidence in procedures. It provides an opportunity for participants to apply theory into practice, and to gain experience that would otherwise potentially put patients at risk, particularly in emergency situations. In addition, simulation provides an environment where all members of the team can learn with and from one another, with the opportunity for debriefing and reflection.

Duran et al. reported an improvement in self-reported confidence levels amongst Vascular trainees afforded access to simulation, with 86% of trainees surveyed supportive of simulation training (17). Simulation accelerates the acquisition of psychomotor skills, procedural understanding, and facilitates assessment of proficiency (18, 19). Specific to EVAR, Vento et al. confirmed that simulation objectively improved the competence of trainees in performing EVAR, with reductions in total procedure time, total fluoroscopy time, time for contralateral gate cannulation, and volume contrast used when compared to the control group (20).

Endovascular techniques are evolving at a remarkable rate. Combined with decreased training hours and the unstructured nature of opportunistic on-the-job training via the traditional Halstedian apprenticeship model, simulation-based procedural training is a promising avenue. The contemporary challenge of lack of procedural exposure is further compounded by improvements in non-invasive vascular imaging techniques, reducing the opportunities for trainees to perform diagnostic angiograms in order to gain essential wire and catheter handling skills (18). Simulation provides a solution by offering an avenue to learn the key steps required in common as well as more advanced procedures under the supervision of a surgical educator.

### Study Aims

3D printed vascular phantoms have a growing role in the pre-surgical simulation and training of complex endovascular procedures. While there is growing interest in the topic, lack of familiarity with 3D printing technology has resulted in slow uptake.

In developing this methodology, our aim was to create inexpensive vascular phantoms with optimal anatomical, haptic and fluoroscopic fidelity. Through iterative prototyping, we have improved upon the workflow, particularly with regards to speed and cost to ensure that initial investment would not pose a barrier to interested departments.

We demonstrate this workflow using AAA phantoms as an example of how CT angiograms acquired as part of standard care can be converted into patient-specific 3D models. Our work has occurred at 3D Med Lab, Australia's first 3D printing laboratory in a public hospital setting. This work has resulted in hollow 3D printed thoracoabdominal aortas with branches which allow for realistic simulation under fluoroscopy.

**Figure 2:**
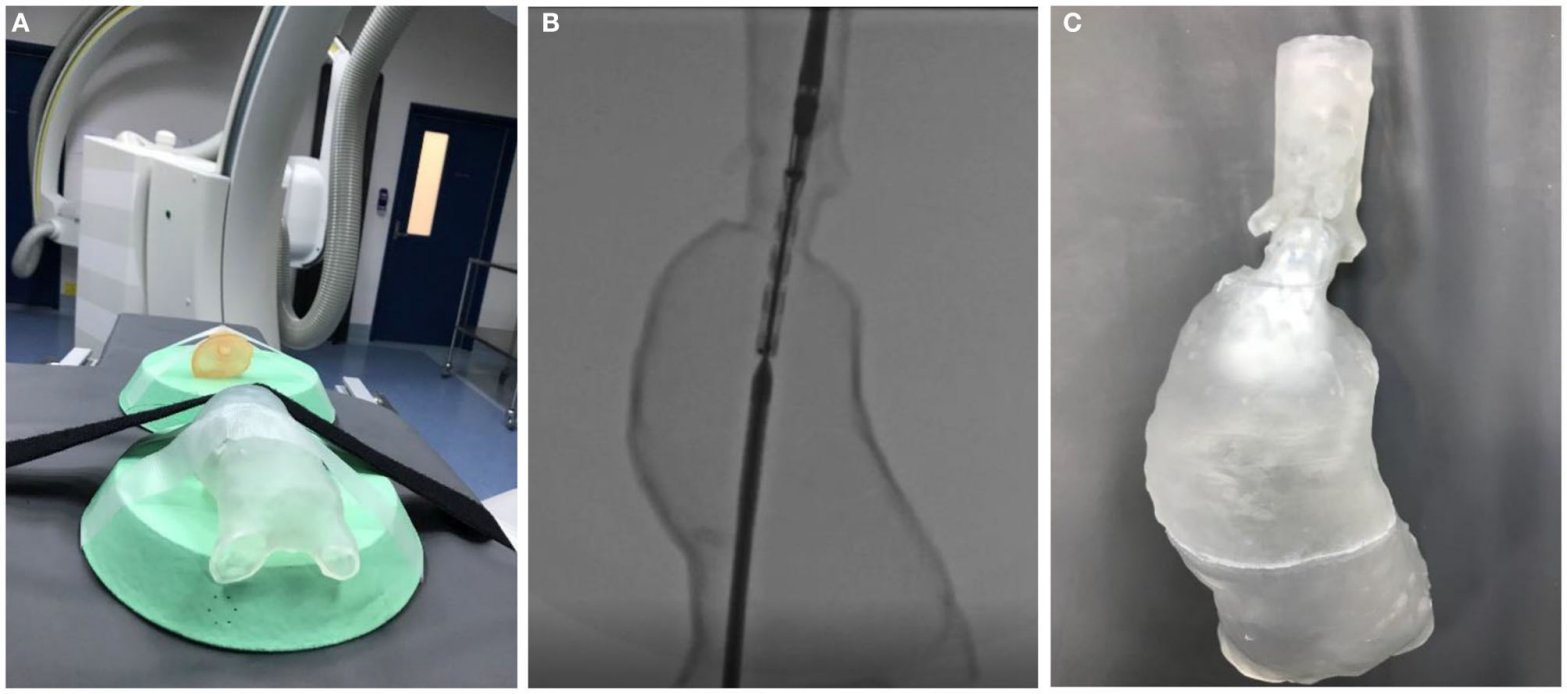
**(A)** A transparent complex juxtarenal AAA phantom being prepared for simulation of a complex fenestrated EVAR procedure. The model was 3D printed with SLA technology using a transparent resin. **(B)** The AAA phantom under fluoroscopy. **(C)** The AAA phantom after deployment of a fenestrated stent graft.

These models, despite current material limitations, successfully mimic the cannulation and deployment challenges encountered during live endovascular surgery (Figure 2). As dimensional and representational material validity is improved, these AAA phantoms have the potential to serve as a powerful adjunct to how complex EVAR cases are planned. In addition, as these models do not degrade, they serve as a valuable tool to simulate EVAR for vascular trainees, as well as to counsel patients as part of the therapeutic relationship.

We seek to inform on the readiness of current levels of 3D printing technologies for the vascular proceduralist, and the feasibility of implementing 3D printing in the hospital setting. A readily reproducible, descriptive methodology for the creation of vascular phantoms has yet to be described in the literature, leading many groups to rely on commercial third parties to create and 3D print their vascular models. Not only would 3D printed vascular phantoms be more economical to produce within the health service setting, it allows for translation to the angiography suite with the efficiency that surgeons are accustomed to.

In summary, our aim was to develop a low cost, low complexity, CT angiogram to 3D printed vascular phantom workflow that could be easily adopted by other groups using open source segmentation packages and inexpensive, commercially available 3D printers.

## Materials and Equipment

### Segmentation Software

There is an ever-expanding selection of medical image processing software available, both commercial and open source. Commercial software utilized by groups in the literature include Mimics (version 23.0; Materialize NV, Leuven, Belgium, 2020), InVivoDental (version 6.0; Anatomage, San Jose, CA, 2020), OnDemand3D (APP version 1.0; CyberMed Inc, Seoul, Korea, 2020), and OsiriX Imaging Software (version 11.0; Pixmeo, Geneva, Switzerland, 2020). However, licensing fees commence at thousands of dollars, which can be challenging for surgical units to justify at the outset.

**Figure 3:**
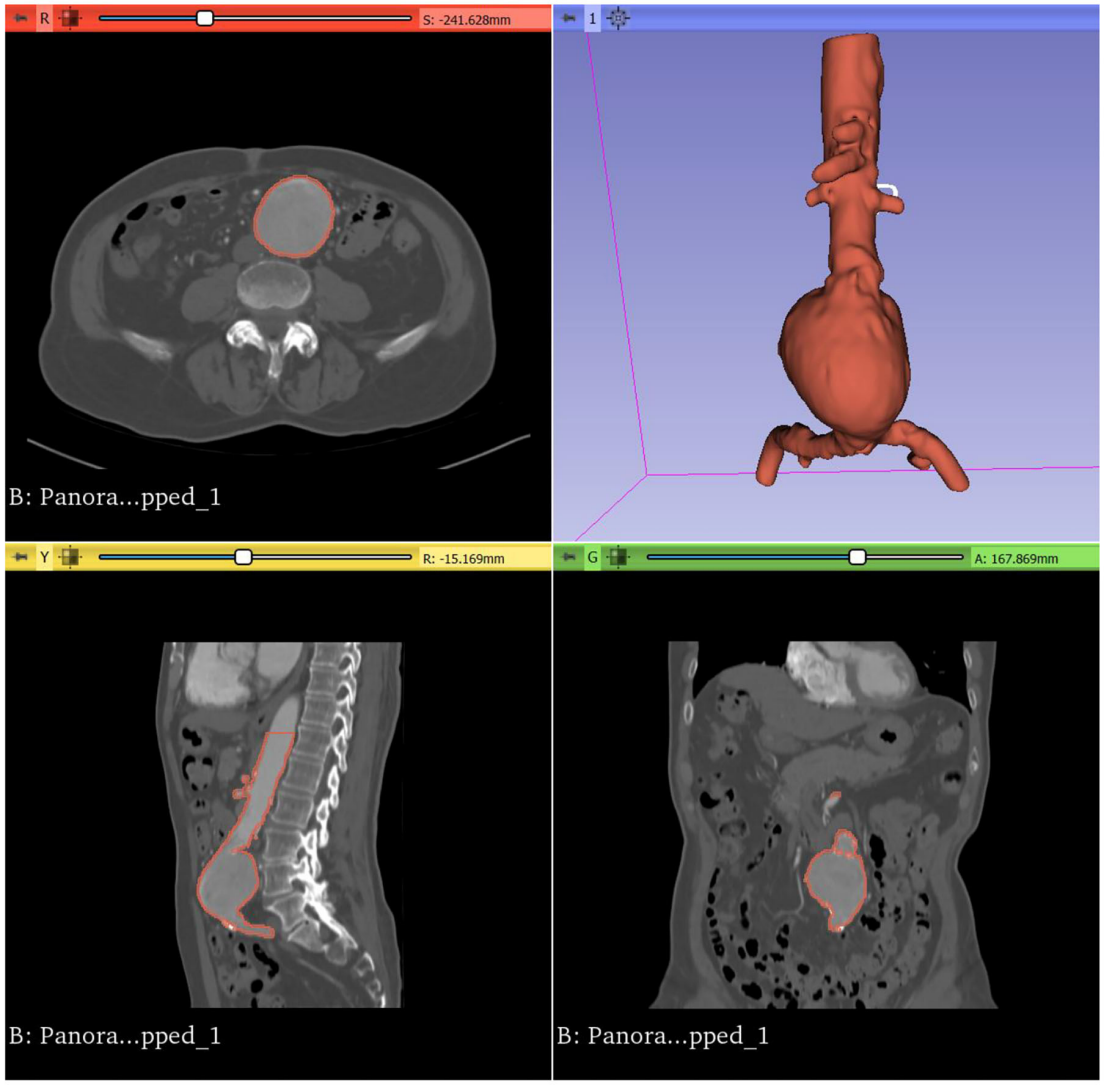
Surface model of an infrarenal AAA viewed in 3D Slicer.

Open source software such as 3D Slicer (version 4.11; Harvard, US, 2020), and ITKsnap (version 3.6; Pennsylvania, US, 2020) present accessible alternatives. Most of our group's experience has been with the open source software 3D Slicer (21), a platform for the analysis and visualization of medical images, available for download at https://www.slicer.org/ (Figure 3). The user interface of 3D Slicer is modular in nature, with powerful plug-in capabilities for additional algorithms and applications.

### 3D Printing Technology

The most readily available 3D printing technologies for the vascular proceduralist include those accessible via university links, or those sufficiently affordable and compact to be located within the hospital setting. The most common 3D printing modalities include Fused Deposition Modeling (FDM), Stereolithography (SLA) and Inkjet techniques.

FDM is the most accessible 3D printing technique for those interested in experimenting with the technology, retailing for as little as a few hundred dollars. The technology involves the building of layers via the extrusion of heat-softened polymers. Common materials include the rigid thermoplastics Acrylonitrile Butadiene Styrene (ABS), Polylactic Acid (PLA), as well as flexible thermoplastics such as Thermoplastic Polyurethane (TPU) and Thermoplastic Elastomer (TPE), soft, rubber-like filaments. FDM has been used to 3D print surgical guides (22), patient-specific anatomical models for presurgical simulation (23), and even patient-tailored pharmacotherapeutics at individualized doses (24).

SLA utilizes an ultraviolet laser, which is selectively scanned over a vat of photo-active photopolymer, curing and solidifying specified areas on the surface of the liquid. As the process continues, the final object is built up layer by layer. Thus far, SLA has been used for anatomical modeling in presurgical simulation (25) and training (26), and in the creation of scaffolds for tissue engineering (27).

Inkjet 3D printing is an extension of the conventional two-dimensional paper printing technique. Hundreds of microscopic nozzles selectively deposit droplets of photopolymer one layer at a time, which are flash cured using a UV lamp. Retailing for hundreds of thousands of dollars, inkjet printing is the most expensive of the 3D printing techniques described, requiring collaboration with academic centers at the outset until its expense can be justified. This technique allows for multiple anatomical structures to be 3D printed in one piece, allowing for basic discrimination between tissues for the purposes of simulation (28, 29).

We provide interested vascular proceduralists with examples of aortic phantoms 3D printed with each of these technologies, as well as our experiences regarding their suitability for common applications (Figures 6–8).

**Figure 4:**
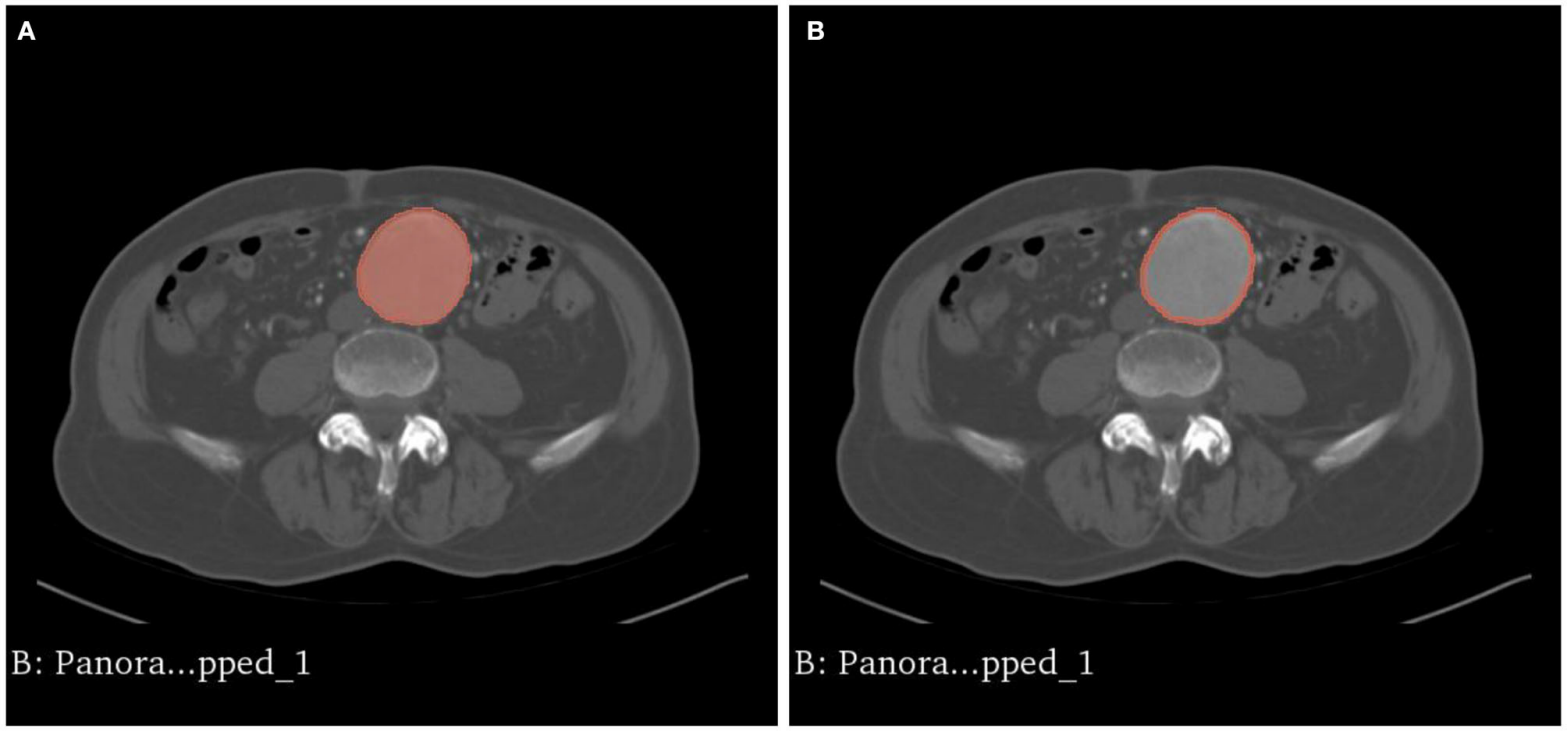
**(A)** The intraluminal contrast within the AAA is highlighted. **(B)** The model is dilated outwards to approximate the external surface of the AAA, resulting in a hollow phantom.

**Figure 5:**
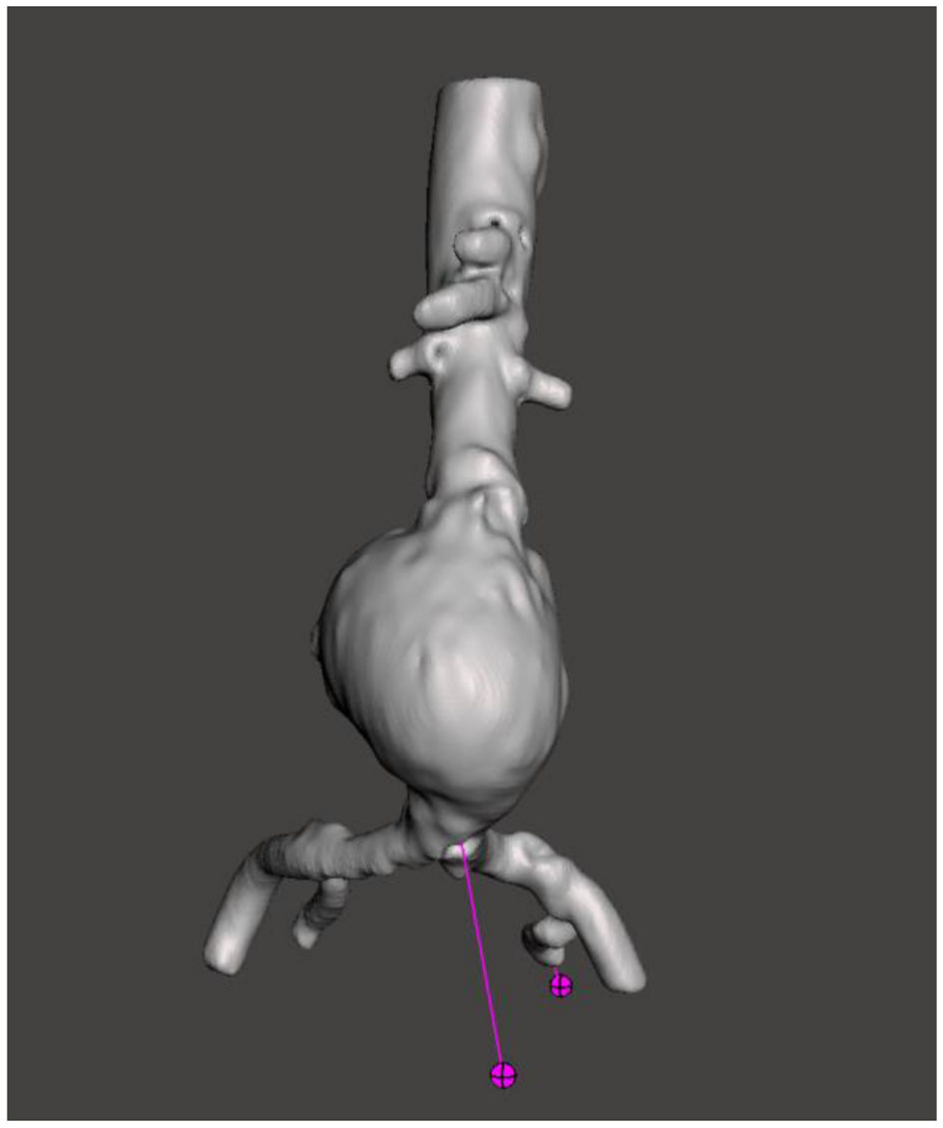
Using Meshmixer, mesh defects in the AAA phantom are identified for repair prior to finalizing the model for 3D printing.

**Figure 6:**
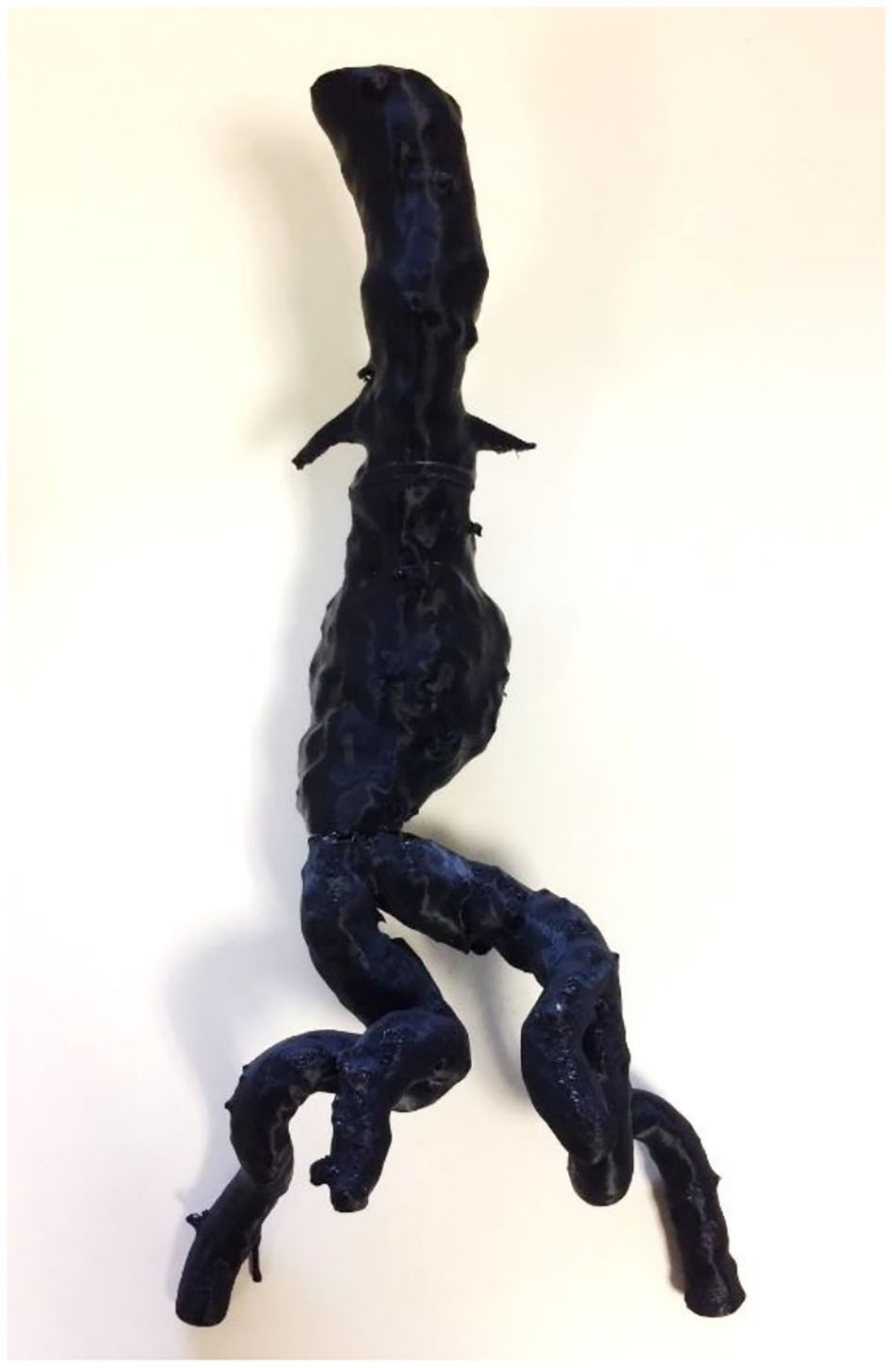
A complex juxtarenal abdominal aortic aneurysm 3D printed for presurgical planning using FDM technology. In addition, the 3D printed model was used to plan the fenestrations of a commercially produced endograft.

**Figure 7:**
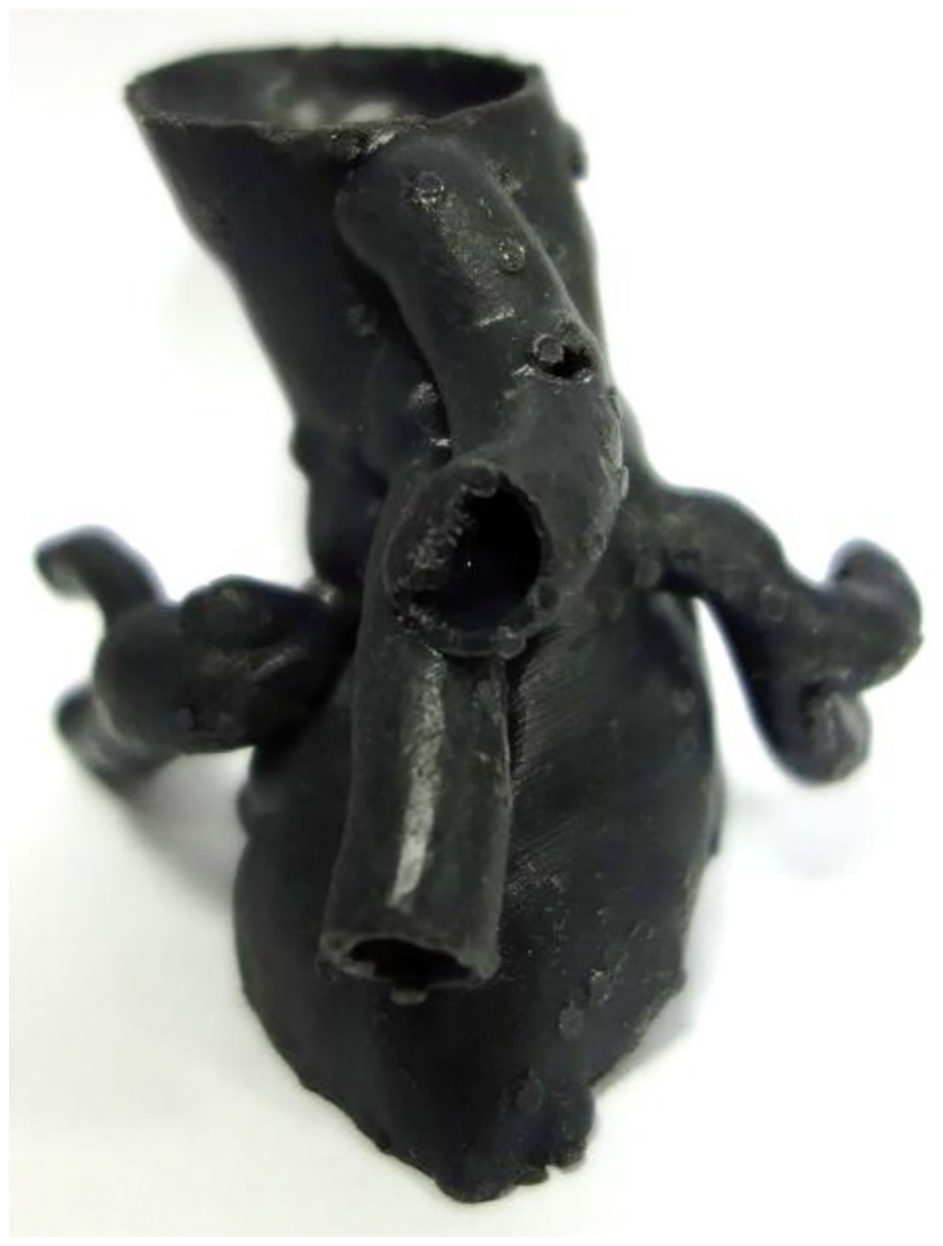
The visceral segment of a complex juxtarenal AAA 3D printed in flexible resin using SLA technology for presurgical planning.

**Figure 8:**
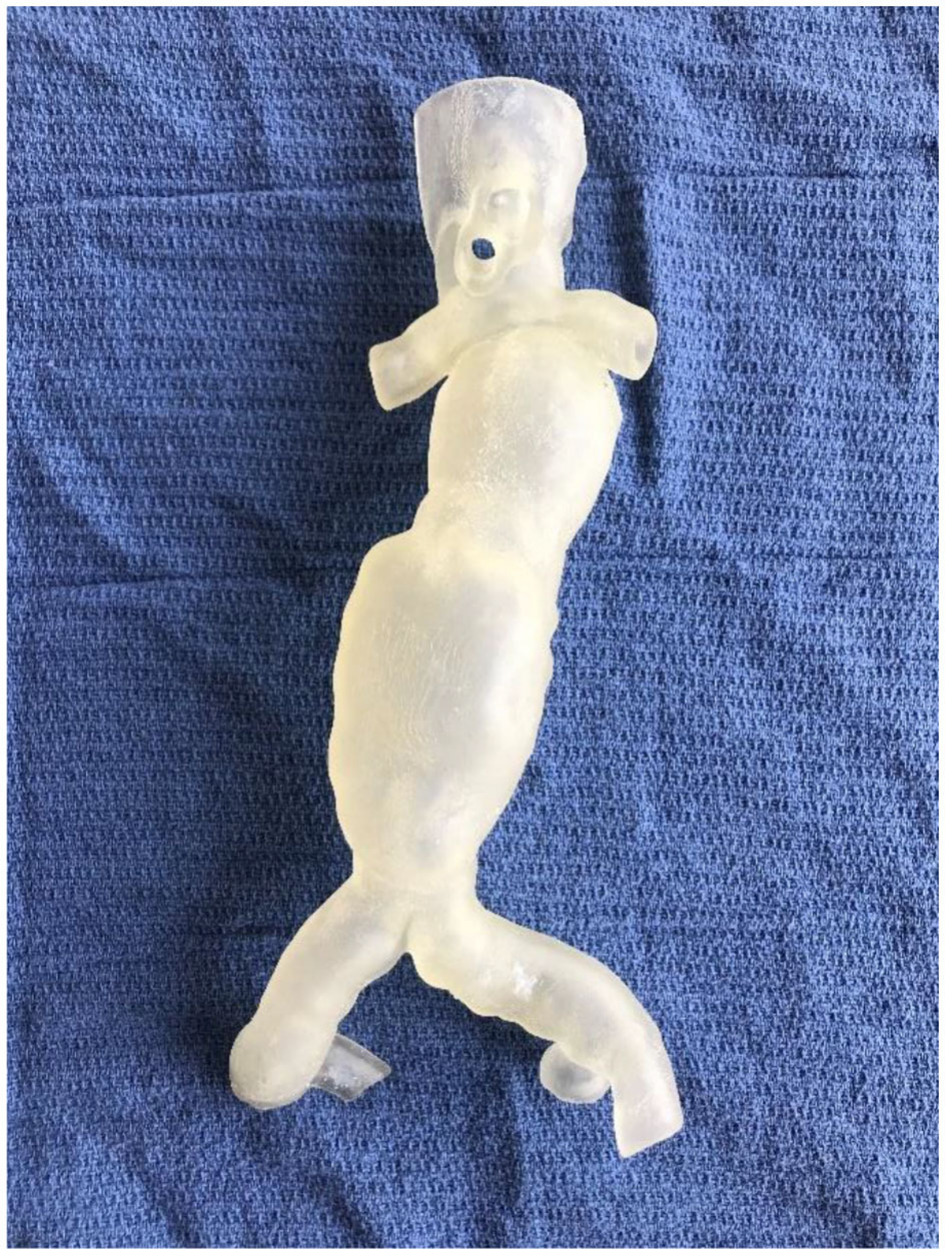
A flexible, hollow, translucent AAA phantom 3D printed using Inkjet technology.

## Methodology

We describe our methodology for isolating regions of patient vasculature to generate surface models for 3D printing, however the vascular models generated can equally be viewed on a tablet, through a Virtual Reality headset, or projected onto a screen for viewing.

Using 3D Slicer, CT aortograms were converted to representative patient-specific AAA models. The models were then prepared for printing using another open source computer aided design (CAD) software, Meshmixer (version 3.5, Autodesk, California, US, 2020), and 3D printed using a variety of 3D printing techniques in order to assess their suitability as aortic phantoms (Figures 4, 5). Depending on the 3D printing technology used, these models cost AUD$20–1,000 and were produced in 12–48 h.

### Image Acquisition

CT aortograms acquired as part of AAA surveillance or preoperative workup are an ideal starting point for this workflow, with intraluminal contrast greatly facilitating the isolation of vascular anatomy. Due to its increasing availability and ever-improving speed and quality, non-invasive CT angiography has become the conventional imaging modality for visualizing vascular anatomy and pathology (30). The accuracy of image processing is highly dependent upon the quality and resolution of the original CT imaging, with slice thicknesses <1 mm yielding superior results.

Although not the modality of choice at our center, Magnetic Resonance Angiography (MRA) presents an ideal alternative, likewise due to the presence of intraluminal contrast. B-mode ultrasound has also been utilized with success in the literature in 3D printing arteriovenous fistulas (31), however ultrasound as a modality is more heavily operator dependent, less readily available in the clinical setting due to staffing demands, and cannot be applied to all vascular territories. A key concept is that the imaging protocol which best visualizes the vascular anatomy for surgical planning will similarly result in the best imaging for 3D printing.

### Generating 3D Models From CT Angiograms

Generating 3D models from patient CT angiography is performed via the process of image segmentation, which involves partitioning an image into multiple segments for meaningful analysis. In the context of CT aortograms, a contrast-enhanced AAA and its branches are efficiently isolated from the surrounding soft tissue by leveraging the concept of Hounsfield Units.

Using the *Segment Editor* module, CT aortogram datasets were automatically segmented using the *Threshold* function. The *Threshold* tool divides the CT angiography dataset into two segments, based on the Hounsfield Unit range selected. This labels the voxels, or three-dimensional pixels, as either colored or uncoloured, covering areas of the selected intensity values throughout all slices of the CT angiogram.

Segmentation via thresholding is performed based on intensity values alone. Hence the process, whilst automated, may result in artifact, or unwanted areas that have been highlighted due to their similar density to the region of interest. These can be removed using the *Save Island* function. *Save Island* retains the selected anatomy and removes disconnected voxels with the same intensity.

### Creating a 3D Model

In 3D Slicer, a preview of the segmented AAA can be visualized in the 3D viewing screen by toggling *View 3D*. For the purposes of EVAR planning, the AAA can be cropped superior to the visceral segment of the aorta, and inferior to the bifurcation of the common iliac arteries by using the *Scissors* function. The *Hollow* function converts the external surface of the intra-luminal contrast into the internal surface, growing a wall of the specified thickness around it, preserving the diameter of the lumen. Once the process is complete, the AAA model is E*xported* to be prepared for 3D printing. The AAA model is by default saved in Visualization ToolKit (VTK) format. This is best converted to Standard Triangle Language (STL) format which is compatible with 3D printers and Computer Aided Design (CAD) software.

### Preparing the AAA Phantom for 3D Printing

Depending on the presurgical simulation intended, or the lesson plan in mind, the model can be further modified using CAD software prior to 3D printing. The vascular phantom can be made modular to introduce increasing levels of complexity, or the inflow and outflow modified to allow compatibility with a fluid circuit. For these purposes, Meshmixer (version 3.5, Autodesk, California, US, 2020) is the open source CAD software favored by many groups in the literature, due to a useful feature in the *Analysis* menu. The *Inspector* tool allows the user-friendly *Auto Repair* of defects in the model prior to 3D printing.

### 3D Printing the AAA Phantom

Once the STL file is ready for 3D printing, it is loaded into the proprietary 3D printing software associated with the 3D printing machine and printed. Phantoms produced using the three main 3D printing modalities, Fused Deposition Modeling (FDM), Stereolithography (SLA) and Inkjet are described below in our *Results*.

## Results

In our experience, 3D printed AAA phantoms are of limited utility in the presurgical planning of standard infrarenal AAA cases. However, they have influenced surgical decision making and device selection in complex cases. In addition, these models have been 3D printed and demonstrated to be useful in a variety of settings, including patient education and engagement, surgical and anatomical education, as well as intraoperative visualization.

There remains room for improvement in the manufacturing of these models, in particular for greater cost efficiency and material properties mimicking those of a diseased aorta as we seek to create a AAA phantom with optimal anatomical, haptic and fluoroscopic fidelity.

### Fused Deposition Modeling (FDM)

FDM is an accessible avenue to begin 3D printing vascular phantoms. There is a large variety of makes and models available on the market, with our group having experience with the Makerbot Replicator 2X (Stratasys, Minnesota, USA), Flashforge Creator Pro (Zhejiang, China), Prusa I3 MK3S (Prague, Czech Republic), and Ultimaker S5 (Utrecht, Netherlands). These hobbyist 3D printers retail in the range of hundreds to thousands of dollars, making them an inexpensive option to begin exploring the technology. Professional tier FDM 3D printers are more reliable and require less maintenance.

The machines consume inexpensive thermoplastics, retailing from $30–80 for a kilogram, which equates to $10–20 per AAA model. Although transparent thermoplastics exist, the resolution of FDM technology and the layered deposition results in at best a translucent end product, as reported by Chung et al. (32). Depending on the size of the AAA being printed, FDM machines require 24–48 h to 3D print the final product, with additional time required for support structures to be manually removed.

### Stereolithography (SLA)

SLA printers retail in the range of thousands of dollars. Most of our experience has been with the Formlabs Form 2 (Somerville, Massachusetts, USA) SLA printer. The benefit of the SLA printer over FDM technology is in its greater resolution, allowing for the creation of transparent 3D printed AAA models which allow the trajectory of devices to be visualized during simulation. 1 L of clear or opaque resin retails for $150, equating to roughly $50–100 per model. Despite the slightly higher cost, we have come to rely on the SLA printer due to its lower print failure rate and ability to create transparent models.

In addition, Formlabs carry an autoclavable dental resin which has received FDA approval to be autoclaved. This has resulted in early work in the literature of 3D printed AAA visceral segment models for the planning of fenestrated physician modified stent grafts on the sterile back table (33).

### Inkjet

Inkjet printers produce multicolored, multi-material models with variable shore hardness. Our group have used the Object500 Connex3 Polyjet (Stratasys, Minneapolis, USA), the Stratasys J750 (Stratasys, Minneapolis, USA), and the Projet 3500 3D printers (3D Systems Corporation, Rock Hill, USA) to produce flexible, translucent aortic phantoms. Due to the greater size of the print bed on these commercial 3D printers, even most large AAAs can be 3D printed in a single piece, costing $700–1,000 in materials.

At the outset, Inkjet printers require a significant financial investment or collaboration with an academic center when building a hospital-based 3D printing service. For example, the Stratasys J750 retails for $600,000, and a 1 kg cartridge of the resin required for 3D printing costs $2,000.

### Postprocessing

**Figure 9:**
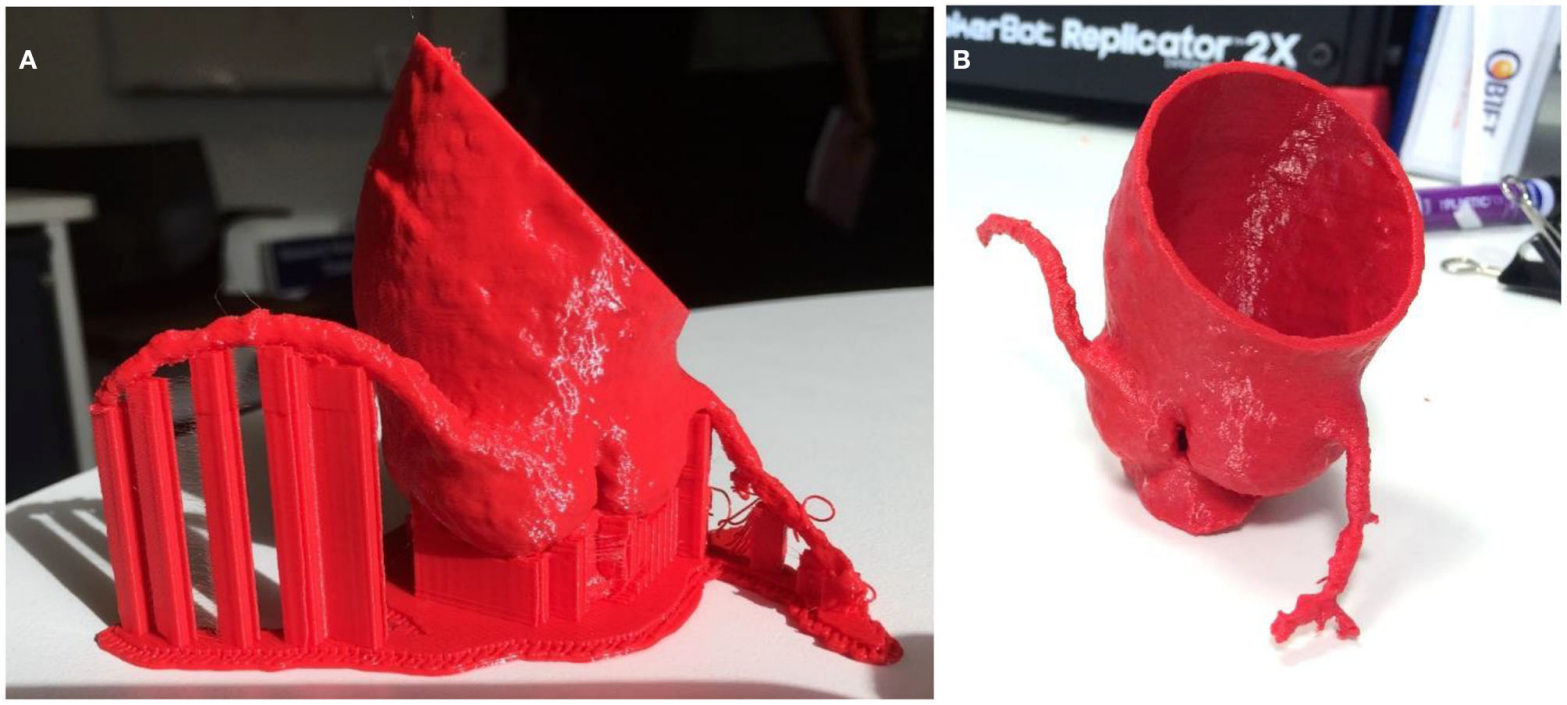
Aortic root model 3D printed in ABS using FDM technology **(A)** prior to removal of supports **(B)** with supports removed.

**Figure 10:**
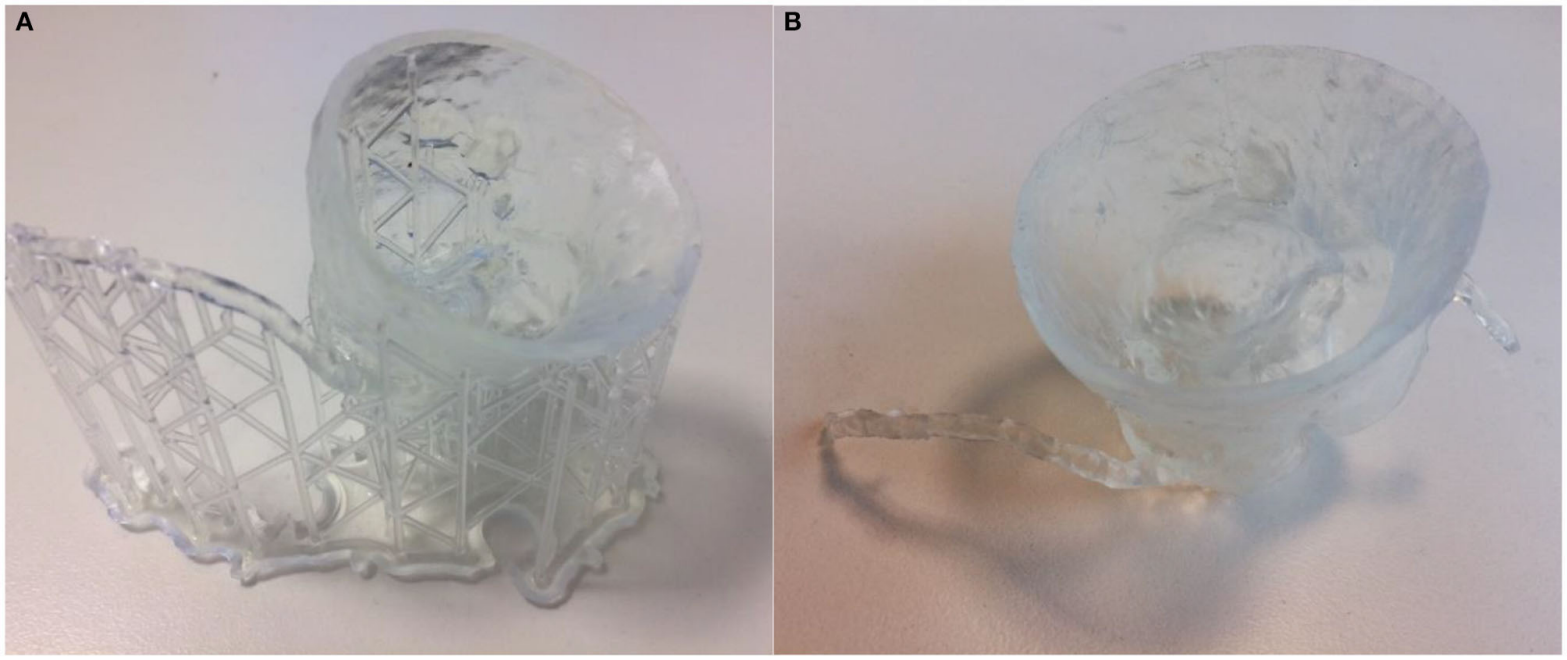
Same aortic root model as previous 3D printed in transparent resin using SLA technology **(A)** prior to removal of supports **(B)** with supports removed.

Regardless of the 3D printing technique, FDM, SLA, and Inkjet 3D printed phantoms require postprocessing prior to use, and the removal of support material required to support the weight of the object during the 3D printing process. Disposal metal scissors and forceps allow for the removal of support material with accuracy and control. This process typically requires a few minutes of manual removal, and up to 20 min for more intricate models (Figures 9–10).

**Figure 11:**
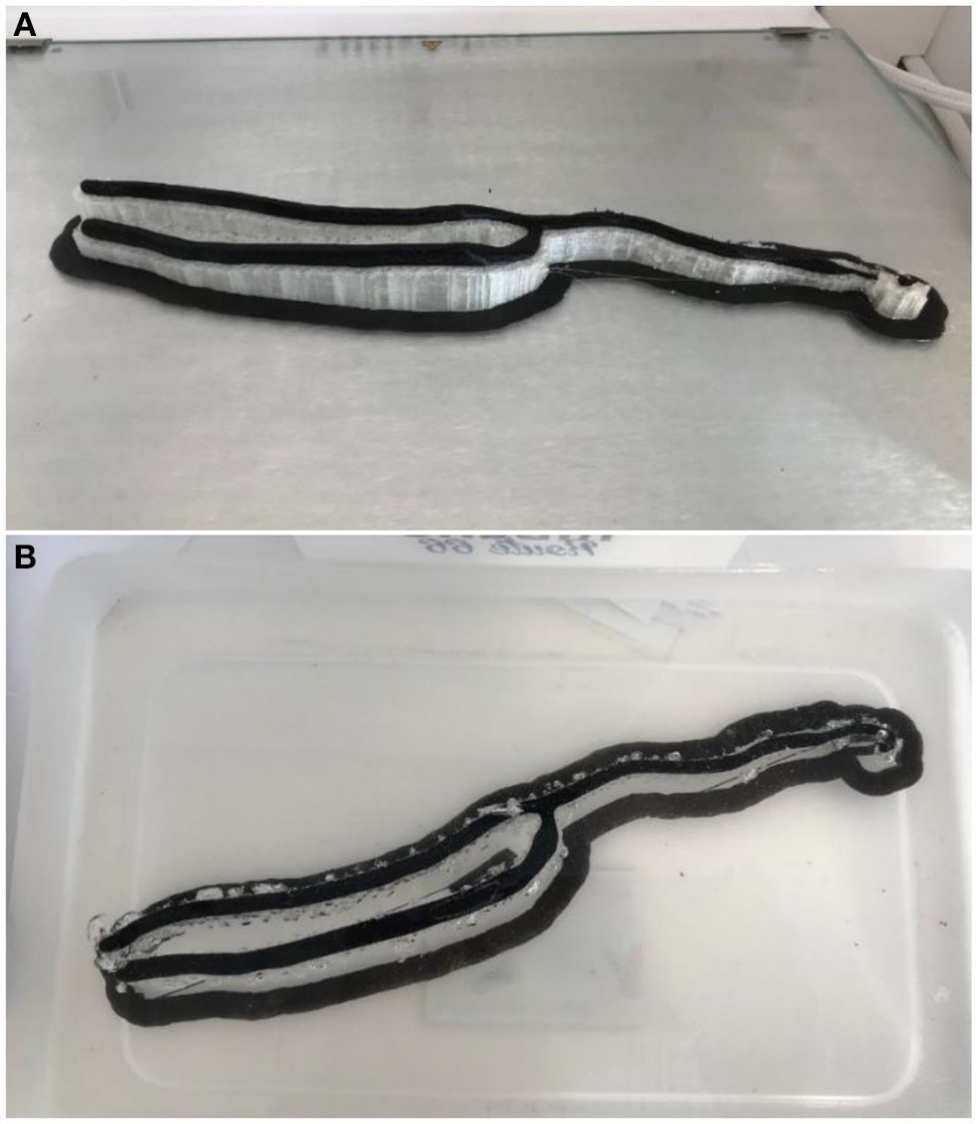
**(A)** A 3D printed brachial artery model prior to removal from the print bed, with supports *in situ*. **(B)** The same model in a water bath to dissolve the PVA supports.

Dissolvable support material is an alternative, with Polyvinyl Acetate (PVA) support material available for dual-extrusion FDM machines, which is readily dissolved in water. Chemical solvents are required to dissolve the large amount of supports present on Inkjet 3D printed models (Figure 11). This is preferable to water jetting the support material, which risks damaging intricate anatomy, particularly when producing flexible models. A disadvantage of soluble support material is the overnight wait for supports to dissolve, adding to the production time of the workflow.

Depending on the 3D printer used, the AAA may need to be sectioned into several parts to fit the available volume for printing. In particular, conventional hobbyist FDM and SLA 3D printers are limited by small print beds, with larger thoracoabdominal aneurysms required to be 3D printed in pieces and joined together with epoxy resin.

### Performance Under Fluoroscopy

**Figure 12:**
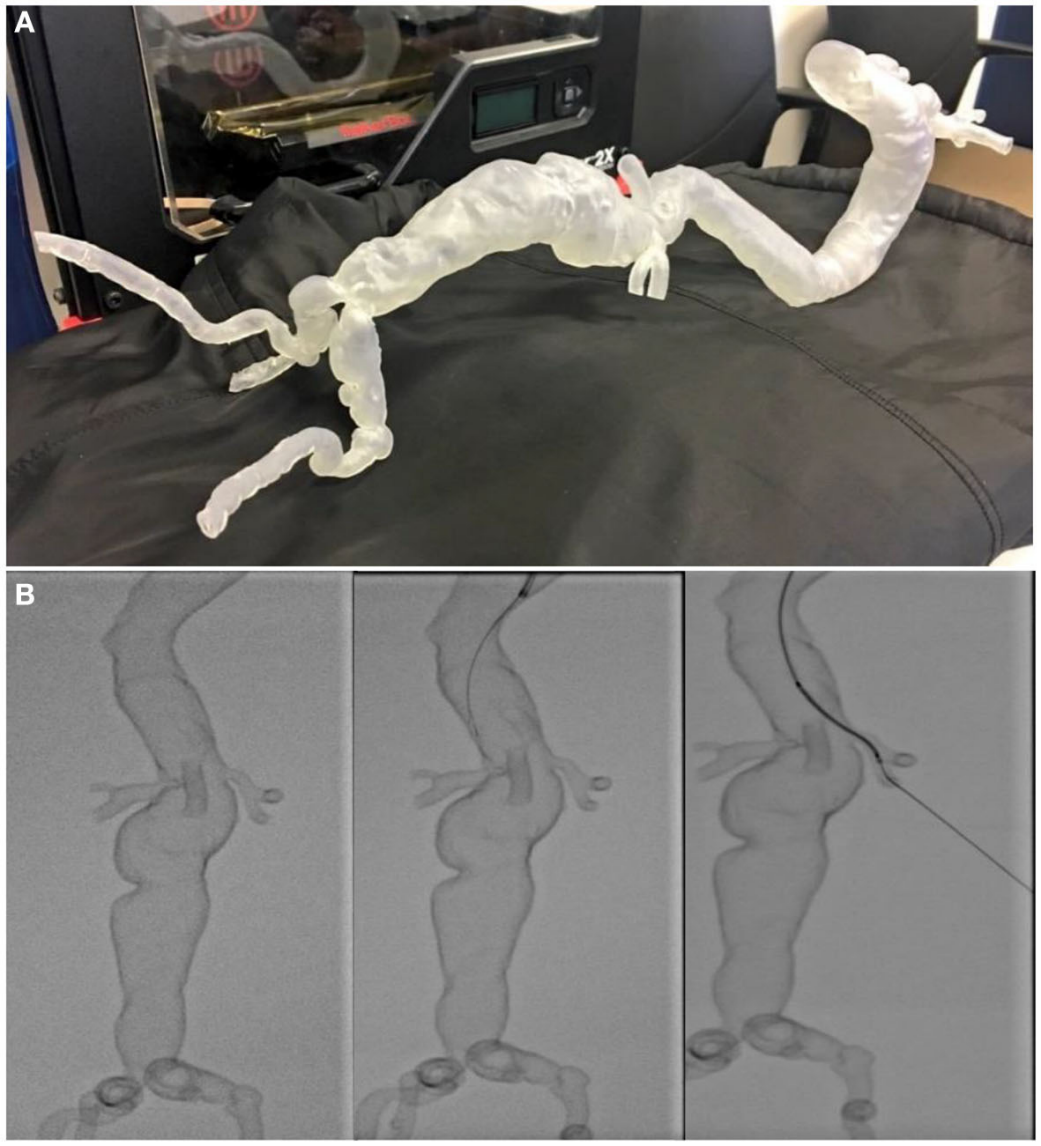
**(A)** A hollow transparent complex thoracoabdominal aneurysm phantom 3D printed using SLA technology. **(B)** Stages of the phantom being cannulated under fluoroscopy for interventional planning. This “pre-flight” simulation was invaluable in predicting navigational difficulties, with the trajectory of guidewires and devices matching what was encountered during live surgery.

FDM, SLA and Inkjet 3D printed AAA phantoms are equally visible under fluoroscopy, allowing for another level of realism to be added to the simulation task. Despite the fact that all three 3D printing techniques performed equally from a visual perspective, due to the resolution of FDM technology which features building melted layers of plastic to create the final product, the grooves between layers were haptically perceptible when traversed by wires (Figure 12). While superior to conventional CT angiography or workstation 3D reconstructions, uneven ridges have the potential to affect the trajectory of guidewires and devices during preoperative simulation. In addition, we have found that the lack of compliance in rigid 3D printed materials adds an additional level of difficulty when traversing tortuous iliac arteries.

## Discussion

AAA phantoms 3D printed using a variety of 3D printing technologies, despite material limitations, successfully mimic the cannulation and deployment challenges encountered during live endovascular surgery. As dimensional and representational material validity improves, they have the potential to serve as a powerful adjunct to how complex EVAR cases are planned.

### Comparison of 3D Printed AAA Phantoms to Other Vascular Territories

3D printed AAAs, and other large vascular phantoms involving the aorta, result in greater challenges with regard to efficient production. Unless access to commercial 3D printers are readily accessible, 3D printed AAAs must often be produced in pieces and joined together prior to use, due to limitations on the size of the print bed on hobbyist machines.

In the case of simulating procedures in smaller vascular territories such as the Circle of Willis or Internal Carotid Artery it may be wiser to create a negative of the anatomy of the interest contained within a solid 3D printed block. From our experience in these smaller vascular territories this creates a more durable result in these small diameter vessels which are more prone to moving or breaking during simulation.

### Anatomical Accuracy of 3D Printed Phantoms

We have outlined the workflow required to produce 3D printed vascular phantoms, with each step introducing a potential avenue for error during the imaging, segmentation, or 3D printing phases. It is evident that further work validating the accuracy of 3D printed models for surgical simulation is warranted. As with all new devices and techniques, surgeons are accustomed to the circumspect application of new technologies to meet the needs of each individual patient.

### Image Acquisition

**Figure 13:**
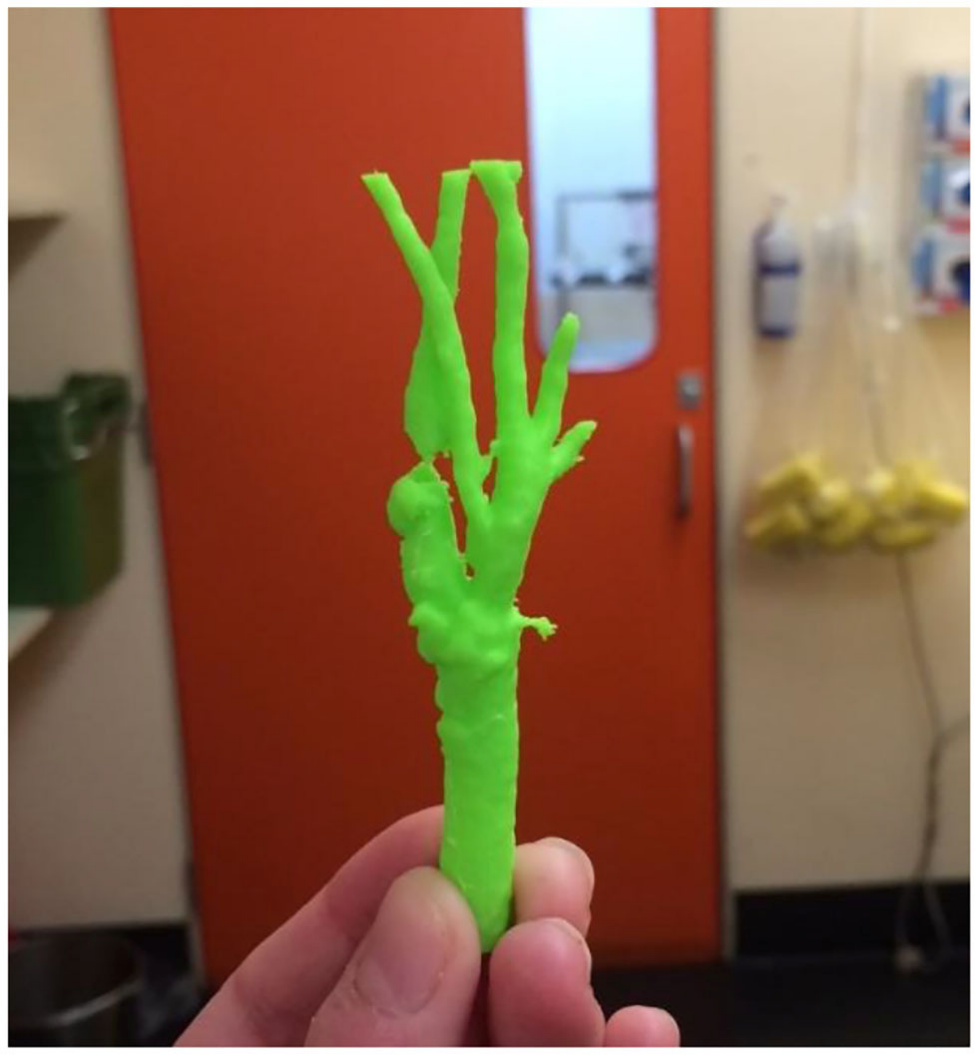
A solid 3D printed internal carotid artery used to plan carotid endarterectomy.

Patient-specific anatomical models are as accurate as the imaging from which they originate. A useful principle is that the imaging modality that best visualizes the anatomy for conventional surgical planning will produce the most detailed anatomical model. Imaging modalities are ever improving, with these improvements benefiting patients regardless of whether an anatomical model is 3D printed (Figure 13). In the vascular territories, optimizing vascular imaging requires the mitigation of motion artifact, and sufficient contrast discrimination and resolution.

For example, dual-energy CT angiography would allow for better contrast discrimination of intravascular calcium from contrast, as well as improving temporal resolution in both peripheral and aortic vascular territories (34, 35). Similarly, electrocardiographic-gated CT coronary angiography allows for the reduction of motion artifacts in valvular heart disease (36), leading to a superior 3D printed anatomical model.

It is evident that as 3D printing becomes more grounded in conventional surgical practice, improving current imaging protocols in order to optimize the final 3D printed product is a necessary area of further study.

### 3D Modeling

Medical image processing software is the next step in the workflow that has a potential to introduce error. Kang et al. compared four of the most readily available commercial software options, InVivoDental (version 5.0; Anatomage, San Jose, CA, 2015), Mimics (version 14.0; Materialize NV, Leuven, Belgium, 2015), OnDemand3D (APP version 1.0; CyberMed Inc, Seoul, Korea, 2015), and OsiriX Imaging Software (version 3.7; Pixmeo, Geneva, Switzerland, 2015) in the creation of craniofacial models (37), determining that InVivoDental was most accurate in producing 3D models from CT imaging.

All four segmentation software packages were accurate at the voxel or subvoxel level; however, there were statistically significant differences in all anatomical regions between the four software packages tested. Kang et al. were not able to access the proprietary coding of these commercial software packages, hence the study was unable to apply the same standardized parameters across the board, limiting direct comparison. Despite this, the fact that all models were accurate to the level of individual voxels was a reassuring finding.

### 3D Printing

There is currently no gold standard for validating the accuracy of patient-specific anatomical models produced by 3D printing. However, early work from multiple groups suggests the accuracy of 3D printed models are within acceptable limits for presurgical planning (38–41).

Hazeveld et al. compared the accuracy of different 3D printing technologies (42). Given what is known about the accuracy of each 3D printing technology, the study confirmed that dimensional error was lowest for Inkjet 3D printing, followed by SLA, and finally FDM. Specific to 3D printed vasculature, Takao et al. determined splenic artery aneurysms 3D printed using FDM printers to be highly precise and accurate, with the cross-sectional areas amongst the 3D printed models within SD <0.05 cm^2^ (range 0.00–0.05) (6).

### Determining Validity

The ideal AAA phantom is one that is not only anatomically accurate but boasts high haptic and fluoroscopic fidelity. In developing this workflow, we have sought to produce 3D printed AAAs with the highest possible representational validity within the constraints of a bench to bedside approach, accessible to the hospital vascular proceduralist.

The necessity of each measure of fidelity depends upon its intended application. For example, for the purposes of presurgical simulation and the education of trainees, high haptic and fluoroscopic fidelity are much more important than when a 3D printed AAA model is used to educate patients. As previously discussed by our group, the validity of a simulation tool can be evaluated in several ways, with a current lack of objective evidence as to how the different measures of validity can be assessed (43). Face validity involves comparing the anatomical and haptic fidelity of a simulator to the current “gold standard” trainers or to performing the procedure *in vivo* (44). It is a highly subjective, but most common measure used in the medical simulation literature. Predictive validity is a much more objective and desirable goal, and involves an assessment of patient outcomes (44) as a result of the simulation. As previously discussed, there are clinical trials underway exploring if 3D printed anatomical models for presurgical planning do indeed improve patient outcomes (1), but none on the topic of endovascular intervention.

### A Hospital-Based 3D Printing Service

3D printed anatomical models present a promising new frontier in the planning and simulation of complex surgical procedures. The American Medical Association has recognized the potential of 3D printed anatomical models in presurgical planning and in producing surgical guides, introducing two reimbursement codes last year (45).

The strength of 3D printing anatomical models when compared to other technologies is its unprecedented accessibility to the vascular specialist, allowing for just-in-time manufacturing. When compared to commissioning an external commercial provider, 3D printed vascular phantoms can be translated from bench to bedside in a matter of hours, lowering costs to surgical units and the time required for the model to be shipped to the hospital. In addition, the current COVID-19 pandemic has emphasized the importance of local health services to be self-sufficient with regard to supplies.

However, the centralized manufacturing approach requires local hospitals to invest in the medical image processing, CAD and 3D printing hardware and skills outlined in this manuscript in order to access this technology. While this would result in significant cost savings for the anatomical models themselves, with flow-on effects of reduced theater time and blood loss resulting in cost savings to the hospital system, it represents a more substantial initial investment.

### Limitations of Current Technology

**Figure 14:**
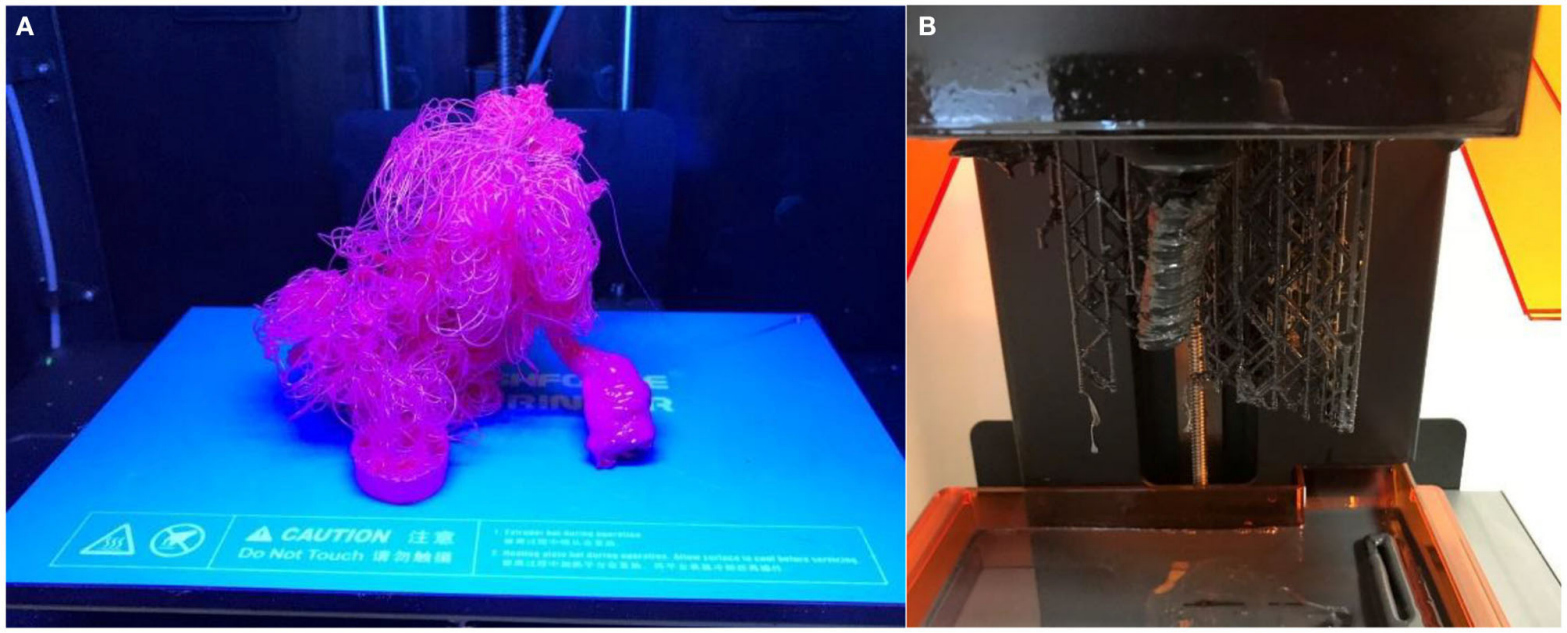
Failed prints on **(A)** FDM. **(B)** SLA 3D printers.

3D printing remains a rapidly evolving technology which is becoming increasingly accessible to vascular proceduralists and the general public. With current levels of technology, inexpensive hobbyist 3D printers are less reliable than their commercial counterparts, particularly when first experimenting with the print orientation of objects. Despite this, they remain an accessible starting point for vascular specialists seeking to begin their foray into the technology (Figure 14). The print failure rate is lessened when printing smaller models, when compared to large vascular phantoms such as thoracoabdominal aneurysms, and when 3D printers are adequately maintained.

## Conclusion

Once the challenges in developing this workflow were overcome, 3D printed anatomical models have become commonplace in the planning of complex procedures in our Vascular Unit and the broader Department of Surgery. Physical patient-specific models have proven to be a valuable addition to standard imaging, and in the rehearsal and modification of devices prior to surgery. When planning the approach to complex AAAs, hollow flexible models are particularly useful for the rehearsal of endograft insertion and positioning via iliac artery access, and in predicting the trajectory of guidewires and devices.

As 3D printing technologies become more common, reliable, and cost effective, their use in preprocedural simulation will flourish. In sharing this methodology, we warmly invite comments from others with an interest in 3D printing in vascular interventional planning in how we can further explore its uses.

## Data Availability Statement

The raw data supporting the conclusions of this article will be made available by the authors, without undue reservation.

## Author Contributions

JC-B contributed to the write up, study design, and experiments performed in this manuscript. DB contributed to the write up and design of this manuscript. JC contributed to the write up, design, and supervision of this manuscript. All authors contributed to the article and approved the submitted version.

## Conflict of Interest

The authors declare that the research was conducted in the absence of any commercial or financial relationships that could be construed as a potential conflict of interest.
